# The effect of pH on the structure of Bluetongue virus VP5

**DOI:** 10.1099/jgv.0.002018

**Published:** 2024-08-20

**Authors:** Hanwen Zhang, Kamel El Omari, Geoff Sutton, David I. Stuart

**Affiliations:** 1 Division of Structural Biology, University of Oxford, Centre for Human Genetics, Oxford, UK; 2 Diamond Light Source Ltd, Harwell Science & Innovation Campus, Didcot, UK; 3 Chinese Academy of Medical Science (CAMS) Oxford Institute (COI), University of Oxford, Oxford, UK

**Keywords:** Bluetongue virus, pH effect, virus entry, virus capsid structure, VP5, X-ray crystallography

## Abstract

The unenveloped Bluetongue virus capsid comprises several structural layers, the inner two comprising a core, which assembles before addition of the outer proteins, VP2 and VP5. Two symmetric trimers of VP5 fit like pegs into two distinct pits on the core and undergo pH conformational changes in the context of the virus, associated with cell entry. Here we show that in isolation VP5 alone undergoes essentially the same changes with pH and confirm a helical transition, indicating that VP5 is a motor during cell entry. In the absence of VP5 the two pits on the core differ from each other, presumably due to the asymmetric underlying structure of VP3, the innermost capsid protein. On insertion of VP5 these pits become closely similar and remain similar at low pH whilst VP5 is present. This natural asymmetry presumably destabilises the attachment of VP5, facilitating ejection upon low pH, membrane penetration and cell entry.

## Introduction

Bluetongue virus (BTV) is transmitted by blood-feeding midges (*Culicoides* sp.), causes haemorrhagic diseases in livestock and is responsible for considerable global economic losses.It is an unenveloped icosahedral virus, belonging to the *Orbivirus* genus of the family *Sedoreoviridae*, with a multilayer capsid enclosing a segmented double-stranded RNA (dsRNA) genome. The inner two capsid layers (composed of VP3 and VP7) comprise a core particle which remains intact following infection, whilst the outer layer contains two proteins, VP2 and VP5. As an unenveloped virus the outer capsid layer is likely responsible not only for cell attachment but also for entry through the host membrane, a poorly understood process in unenveloped viruses. VP2 is the attachment protein and protrudes from the virion surface, whilst VP5 is implicated in membrane penetration.

BTV VP5 is a trimer of molecular weight 177 kDa. There is a considerable body of work aiming to understand how the viruses cross the host membrane in the absence of the membrane fusion mechanism employed by enveloped viruses. Early work implicated acidic pH in BTV entry [1], with further work showing that VP5 has pH-dependent fusogenic activity when expressed on the cell surface [2] and that inhibition of endosomal acidification reduces virus infection [3]. Although VP5 lacks the autocatalytic cleavage site and N-terminal myristoyl group which are the triggers during the entry proteins of reoviruses and rotaviruses, a possible mechanism for VP5 interacting with host membranes came from a paper proposing that it bears two N-terminal amphipathic helices which cause leakiness of the membrane upon low-pH treatment [4]. A major advance came from cryo-EM analyses of the intact BTV virion at pH 8.8 and two acidic pHs, which showed that at low pH there was a conformational change in VP5 which caused it to protrude from the viral surface [5,7]. This work provided strong evidence of, and a reasonably detailed model for, a major pH triggered conformational change in the VP5 trimer, which appears to cause the N-terminal helices to project outward from the particle surface.

Here we characterise VP5 at two pHs by crystallography to provide a detailed analysis of the pH dependent structural changes and consider the consequences at the virion level. This contributes to our understanding of one instance of the relatively opaque membrane penetration process of non-enveloped viruses, where the molecular details of membrane disruption and the structural characteristics of the domains involved are much less well characterised than the membrane fusion processes of enveloped viruses.

## Methods

### Construct design and expression

Eight serotypes (3, 4, 8, 10, 15, 19, 21 and 26) were selected for expression screening in insect cells using the baculovirus system so as to cover the phylogenetic groups reported in [8]. The pOPIN vectors, pOPINE and pOPINF, and protocols developed by the Oxford Protein Production Facility (OPPF) were used to clone full-length VP5 and two N-terminal truncations of 21 and 41/43 amino acids [9]. The N-terminal truncations were designed to remove either one or two amphipathic α-helices which have been shown to have a deleterious effect on protein expression. A total of 54 trial expressions were conducted and after assessing expression levels and solubility BTV-15-VP5-Δ43-Nhis was selected for large scale purification and crystallisation. BTV-15-VP5-Δ43-Nhis, both unlabelled and seleno-methionyl-derivatised [10,11], was purified using metal-chelate affinity chromatography and size exclusion chromatography. Multiangle light scattering was used to characterize purified protein before crystallisation.

### Crystallization

Crystallization trials were performed in a sitting drop vapour diffusion format using 100 nl protein solution adding into a 100 nl reservoir solution set up in 96-well Greiner plates with the Cartesian Technologies robots [12]. Dispensed plates were kept in a 20 °C imager. A total of 14 blocks of different conditions (each block contains 96 conditions) were used for screening for protein crystals. Crystals appeared in both low (pH 6.0) and high (pH 9.0) pH conditions. For the high pH condition, the crystals were formed in an optimal condition of 0.09 M sodium formate, 6.6% PGA-LM and 0.1 M Tris (pH 9.0) and appeared in a week. The rhombic-shaped crystals grew to a size of approximately 100×50 microns. For the low pH condition, the crystals appeared in 2 weeks in the condition of 1.0 M sodium chloride and 0.1 M Tris (pH 6.0). The crystals were mounted in litho loops (Molecular Dimensions, Rotherham, UK) and soaked in mother liquor supplemented with 25% (v/v) ethylene glycol before plunging in liquid nitrogen.

### X-ray data collection

X-ray data collections were carried out at Diamond Light Source. The high pH data from BTV15 VP5 Se-Met crystals were collected on beamline I03 with a Pilatus3 6M detector. Data were collected slightly away from the absorption edge at 0.97918 Å corresponding to an energy of 12 662 eV. Five datasets of 180˚ were collected with a 50μm ×20μm beam size. The rotation per image was 0.1˚. A total of 1800 images were taken for each of the datasets with 0.02 s exposure per image. The crystal belongs to space group P1, unit cell 91.4 Å, 142.2 Å, 142.15 Å; α119.5˚, β96.1˚, γ95.8˚. The low pH data were collected from native crystals on beamline I24 with a Pilatus3 6M detector. Two datasets of 180˚ each, with a rotation of 0.05˚ and 0.02 s exposure per image were collected. All data were processed by HKL2000 [13] and the statistics summarised in Table 1.

### Structure determination

For the high pH structure the Se-Met substructure was solved using SHELXD [14]. The VP5 cryo-EM structure became available at this point and was used as a molecular replacement model and facilitated model building [5]. The anomalous difference map provided further validation that our model is correct. The low pH structure was determined by molecular replacement [15] using the available cryoEM model of the higher pH virus [5].

### Model building / refinement methods

Manual building was carried out in COOT [16] and refinement carried out using PHENIX [15]. Comparisons between structures was performed using Structural Homology Programme (SHP) [17].

## Results

**Fig. 1.**
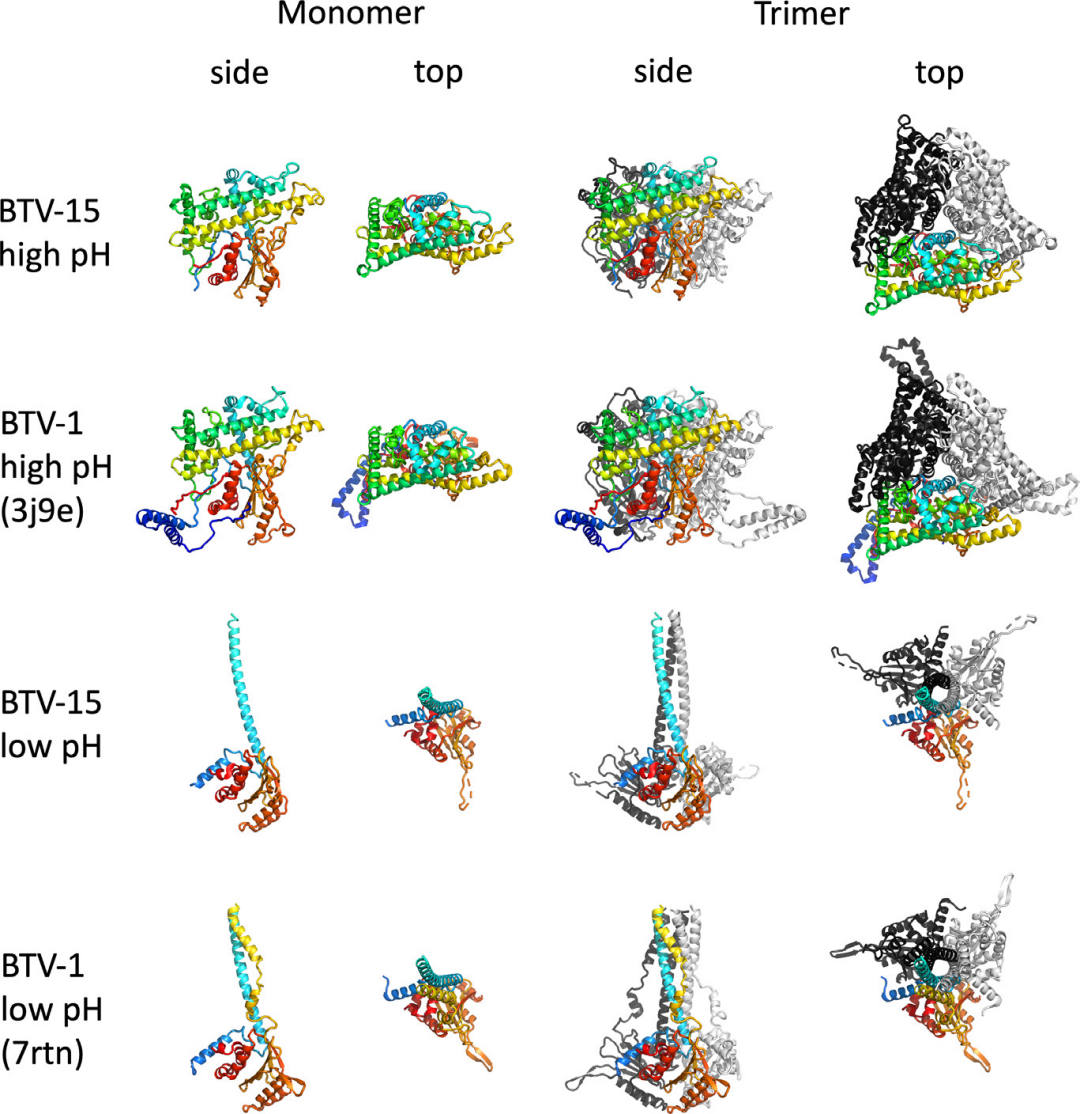
Structures of BTV-15 VP5 high pH, BTV-1 VP5 high pH (pdb 3j9e), BTV-15 VP5 low pH and BTV-1 VP5 low pH (pdb 7rtn). Side and top views are shown for each structure. The monomers and one protomer for the trimers are coloured from blue (N terminus) to red (C terminus) by absolute residue number. The second and third protomers of the trimers are coloured light- and dark-grey respectively.

To enable the expression of a soluble form of VP5 a baculovirus driven expression screen of 48 constructs was performed, covering a range of serotypes and deletions (see Methods). The most promising candidate was a construct of BTV15 VP5 in which the first 43 residues (corresponding to the two N-terminal amphipathic helices) were replaced by a 6xHis tag. This was crystallised in two different pHs, 6 and 9 (Methods). The structure was solved for the high pH form by seleno-methionine labelling, assisted by the contemporaneous release of the structure determined by cryo-EM [18] and refined to 2.8 Å resolution (Methods). The low pH form was solved in the native state and refined at 2.2 Å (Methods). The high pH form, for which 427 out of 484 residues in the construct could be built into the density, is very similar to the structure of BTV1 VP5 solved by cryo-EM to 3.3 Å [5], the sequences show 56% identity and 398 Cαs can be superimposed with r.m.s.d. 0.94 Å (Figs1  2). This crystal form contained nine subunits of VP5, arranged as three trimers, in the crystallographic asymmetric unit. These nine copies were sufficiently similar that satisfactory refinement was achieved using non-crystallographic restraints (Table 1), with average r.m.s.d. between Cαs 0.31 Å.

**Fig. 2.**
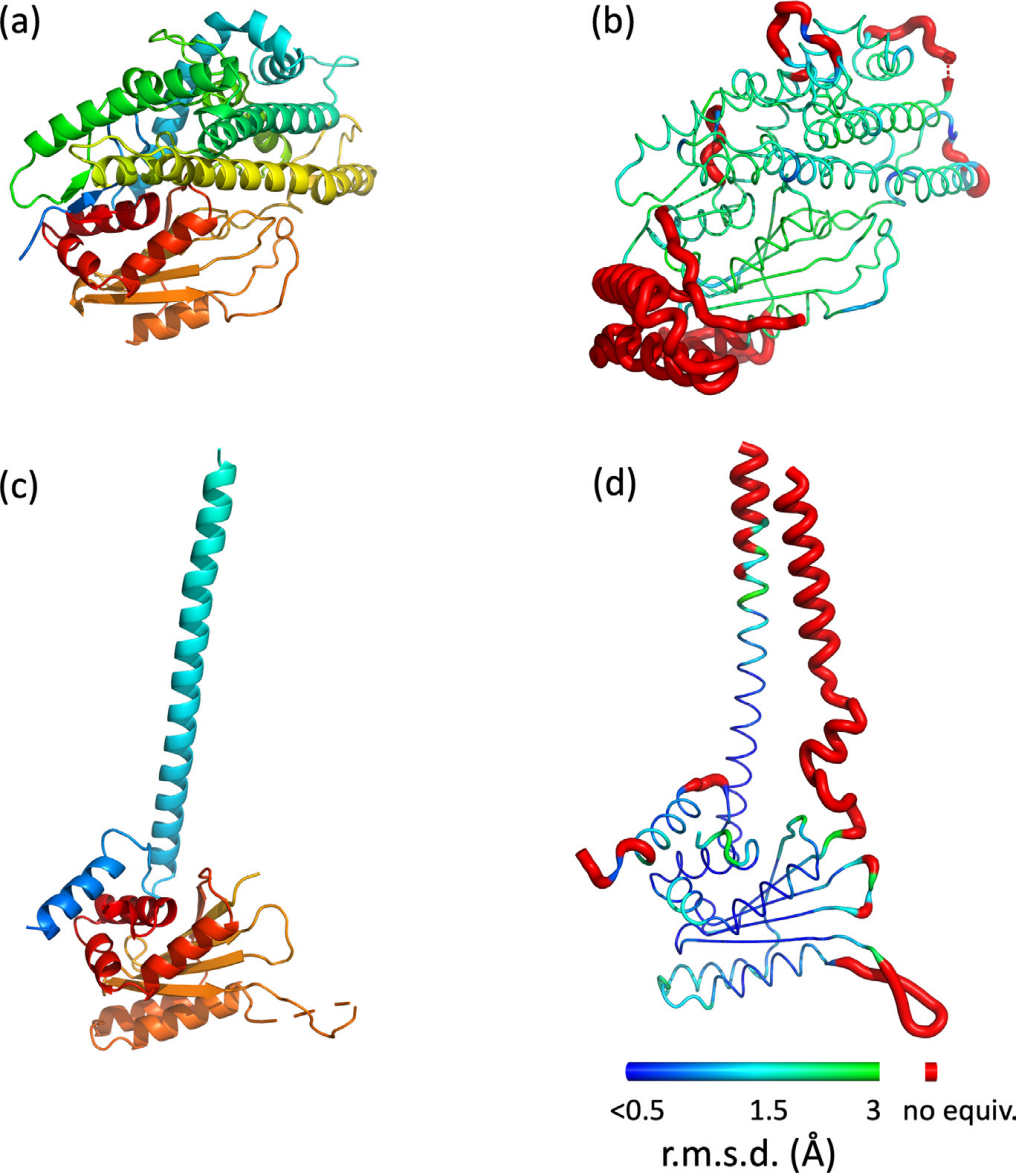
Comparison of the two high pH and two low pH structures. The molecules were aligned with programme SHP [17]. Cartoon representation of the structures of BTV-15 VP5 at high pH (**a**) and low pH (**c**) coloured from blue to red (N- to C-terminus). Putty representation of the structures of BTV-1 VP5 at high pH (**b**) and low pH (**d**). Both colour and worm thickness represent r.m.s. deviation (r.m.s.d.) of equivalent cα atoms (thin, blue: r.m.s.d. <1.0 Å, cyan, thicker: 1.0–2.0 Å, green, thickest: 2.0–3.0 Å). Unaligned regions are coloured red and displayed with exaggerated thickness.

**Table 1.** Data collection and refinement statistics for crsytallographic structures

	High pH (pH9)	Low pH (pH6)
**Data collection**		
**Wavelength (Å**)	0.97918	0.96864
**Space group**	P1	H3
**Cell dimensions**		
a, b, c (Å)	91.4, 142.2, 142.2;	76.9, 76.9, 163.1;
α, β, γ (°)	119.5, 96.1, 95.8	90, 90, 120
**Resolution (Å**)	46.6–2.80 (2.90–2.80)	51.8–2.19 (2.25–2.19)
**Total reflections**	147 513 (15781)	18 627 (12139)
**Unique reflections**	290 549 (30086)	18 479 (1322)
**Rmerge**	0.12 (0.67)	0.07 (---)
**CC50**	0.99 (0.64)	0.99 (0.65)
**I / σI**	11.0 (1.7)	13.6 (2.2)
**Completeness**	98.9 (98.4)	100 (100)
**Redundancy**	3.5 (3.5)	9.9 (9.2)
**Refinement**		
**Resolution (Å**)	46.6–2.80	51.6–2.19
**No. reflections**	147 486	36 944
**No. atoms**		
Protein	30 625	1972
Water/other	---	12
**Rwork / Rfree**	0.22/0.26	0.20/0.21
**Average B-factor (Å^2^)**		
Protein	70	83
Water/other	---	72
**R.m.s. deviations**		
Bond lengths (Å)	0.003	0.003
Bond angle (°)	0.6	0.5
**Ramachandran plot**		
Favoured	94.8%	97.25
Outliers	0.03%	0%

Numbers in parenthesis refer to the highest resolution data shell.

The low pH conformation at 2.2 Å provides a precise description of this state, presumed to be primed for host membrane engagement. Compared to the cryo-EM structure at 3.4 Å resolution [7] the overall structure is very similar (r.m.s.d. 1.22 Å for 194 Cαs) (Figs1  2). Since the two N-terminal helices are deleted from the construct the crystal structure begins at the H4 helix (secondary structure numbering follows that defined for the high pH conformation), which lies against the body of the subunit, following which the chain turns to run upwards away from the body of the subunit in an extended helix, H5 (residues 90 to 137) (Fig. 1). Interestingly the electron density for this extended helix is clear, allowing a reliable model to be built, whereas the density in the cryo-EM reconstruction (EMDB 24684) fades quickly away from the main body of the VP5 subunit, despite which the cryo-EM model is similar to the X-ray structure in this region (Fig. 2).

**Fig. 3.**
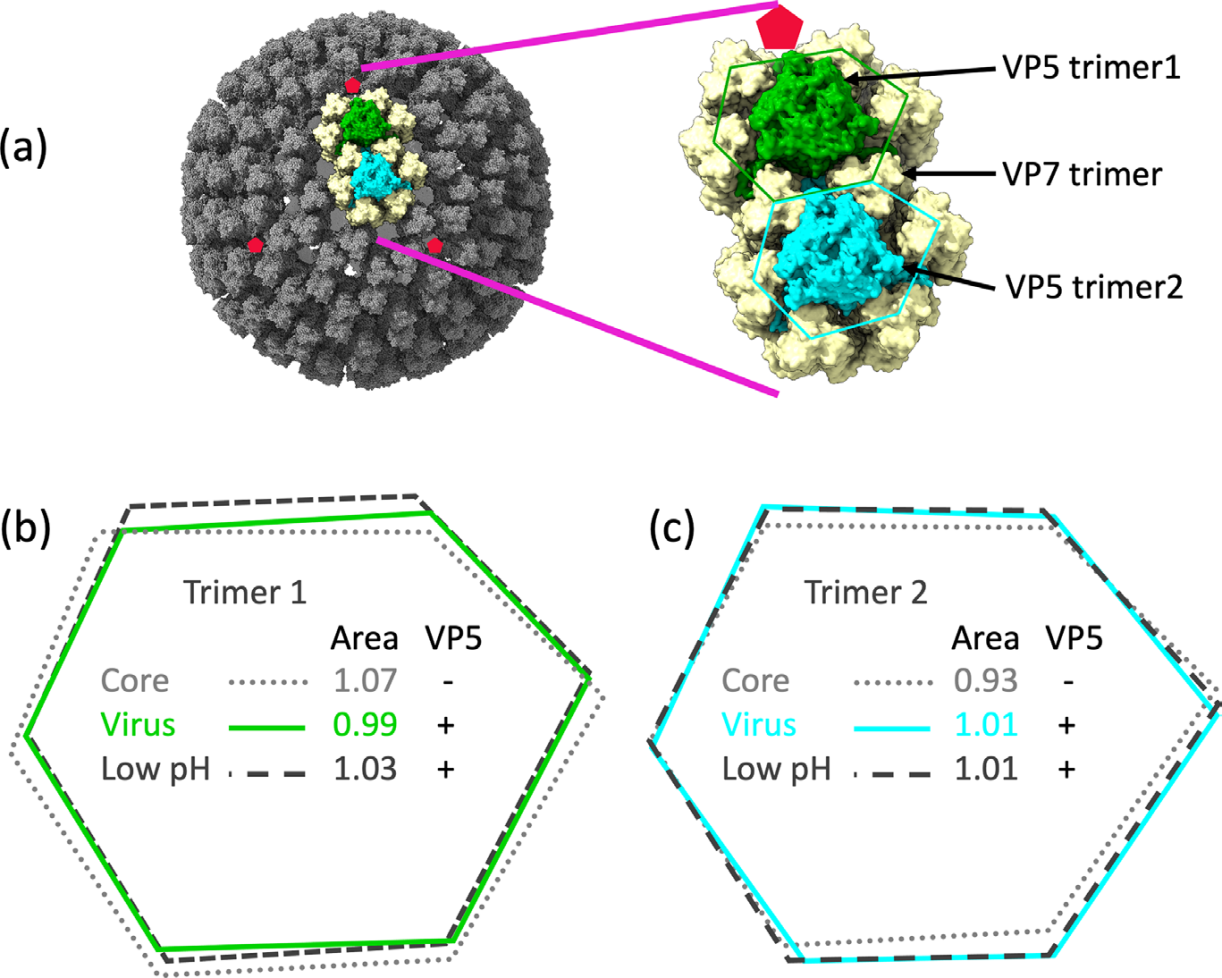
Relative positions of the analysed VP7s (**a**) in the virion with, in more detail, a representation of how the hexagons are defined around VP5 trimer 1 (green) and VP5 trimer 2 (cyan). Relative areas of the hexagons for the core, virus and low pH structures around (**b**) VP5 trimer 1 and (**c**) VP5 trimer 2. The values are normalized such that the average of the areas of trimer 1 and trimer 2 for the virus is 1.00.

In the icosahedral asymmetric unit of the virion there are two trimers of VP5 which fit into pits on the VP7 surface [5,7]. VP7 is arranged in a T=13 lattice with two pseudo hexagonal pits in each asymmetric unit which are surrounded by six trimers of VP7. Each of these pits harbours a trimer of VP5 (Fig. 3a). The fundamental arrangement of VP7 is preserved between the core particle, which contains no VP5, the mature high pH form of the virus and the low pH form of the virus. In the latter two forms the pits are occupied by VP5. Xia *et al.* [7] devised a way of analysing the relative size of the pits, termed (1) and (2) and we have extended this analysis to quantify the structural accommodation of the VP7 trimers on binding the high and low pH forms of VP5.

Atomic models of VP7 and VP3 were fitted into the map of the high pH virus (EMD-6444) using Chimera (University of California San Fransisco). As a guide the BTV core structure (pdb 2btv) was first fitted. Then individual chains of VP3 and trimers of VP7 and VP5 were rigid body fitted with correlation coefficients >0.81. The process was repeated for the low-pH virus (EMD-6445). It became apparent that although the VP3 layer was consistent in all three structures (core, virus, low pH virus), there were some shifts in the positions of the VP7 trimers which altered the sizes of the pits harbouring VP5. To measure the relative positions of the VP7 trimers, and calculate the area enclosed by the hexagons described by the VP7 trimers surrounding each pit (as a surrogate for the cross-sectional area of the pit), hexagons were drawn with each vertex being the centre of the three G199 residues in each VP7 trimer, analogous to the method used in [7]. The area of the hexagon was calculated using Gauss’s area formula [19]. The results are shown in Fig. 3b, c, where it will be seen that in the presence of VP5 the two pits are very similar in size (virus and low pH), whilst in the absence of VP5 (the core) one pit is narrowed and the other is widened compared to the VP5 bound state.

## Conclusion

**Fig. 4.**
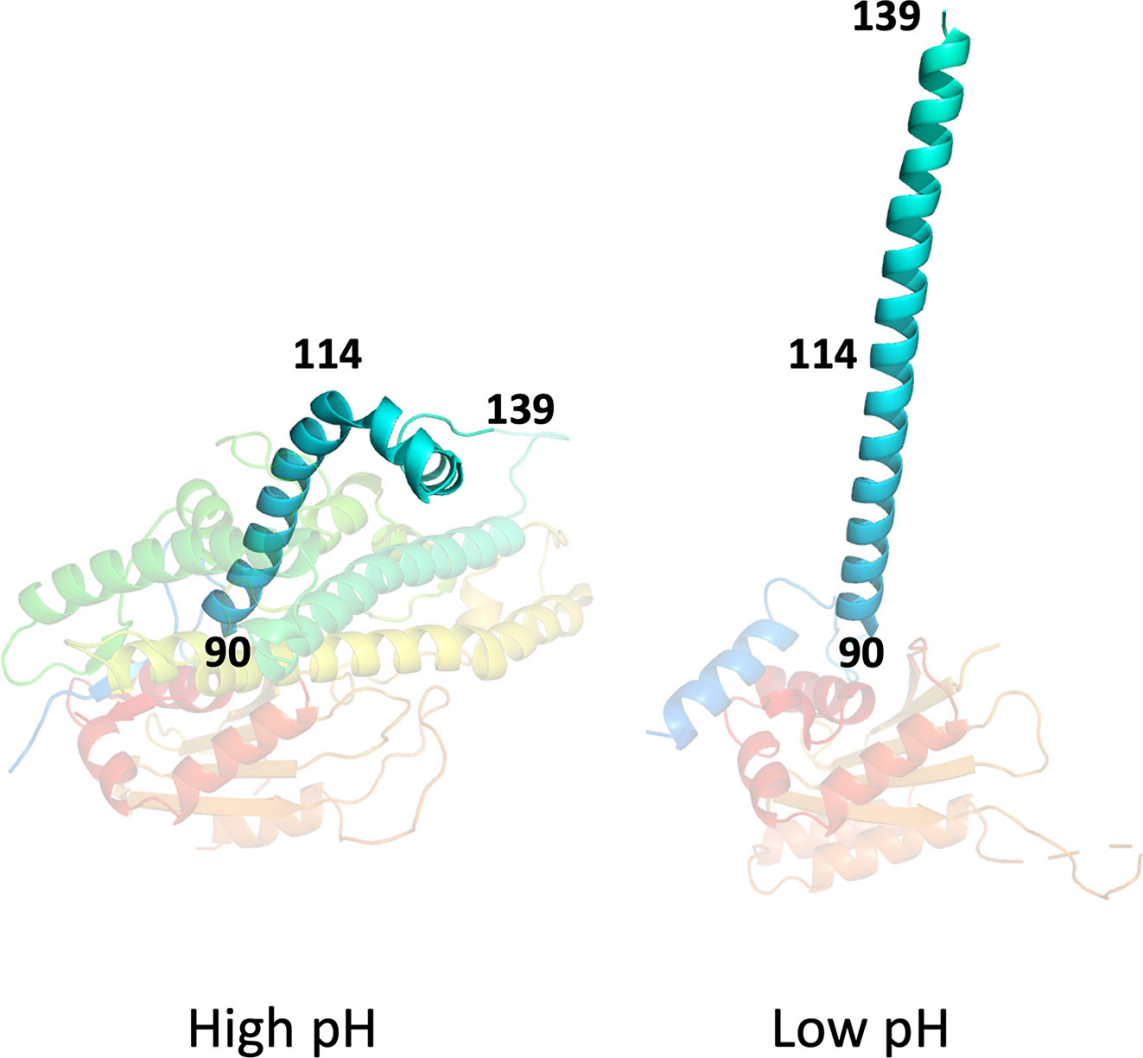
Residues 90–139 in the high pH form undergo a rearrangement to produce the long helix observed at low pH.

Protein VP5 forms part of the outer layer of BTV, with two symmetric trimers of VP5 fitting like pegs into pits on the underlying core. At low pH the trimer undergoes conformational changes in the context of the virus which are associated with cell entry (Fig. 4). Crystals structures of BTV VP5 trimers at high pH (9) and low pH (6) show that at high pH the structure is essentially indistinguishable from that at physiological pH (Figs1  2). Likewise the low pH structure confirms the extended helical protrusion that was inferred but poorly resolved in the previous cryo-EM structure [7]. There are some differences between the structures in the virion and in the crystal (Fig. 2), for instance in the low pH crystal form the so called dagger domain (residues 410–421) cannot follow the path seen in the virus, due to clashes with symmetry molecule in the crystal. In summary we show that VP5 in isolation undergoes essentially the same changes with pH as seen in the virus and confirm the helical transition to produce spikes radiating from the virus surface (Fig. 4). This indicates that VP5 is a pH driven motor during cell entry.

In the absence of VP5 there are two pseudo hexagonal pits on the core, one close to an icosahedral five-fold (peripentonal) and one adjacent to an icosahedral three-fold axis (Fig. 3a). Although these are quasi-equivalent to each other we find that they differ significantly, most likely due to the asymmetric underlying structure of VP3 on to which the VP7 layer adheres (VP3 possesses only T=1 symmetry while VP7 follows T=13 pseudo symmetry, Fig. 3a), thus the cross-sectional area of the hexagonal VP7 array forming the pit is some 14% larger for the peripentonal pit than that adjacent to the three-fold axes (Fig. 3b, c). However upon insertion of VP5 these pits become closely similar (±1%) and change very little at low pH as long as VP5 is present (there is a marginal 2% increase in cross-sectional area). The natural asymmetry of the pits into which the VP5 pegs fit seen in the core will presumably slightly destabilise the attachment of VP5, likely facilitating its ejection at low pH when there is membrane engagement, as part of the process of cell entry. Specifically we expect that this weak attachment allows VP5s to be successively stripped out from the core as the membrane pore enlarges, allowing the core to emerge into the cytoplasm.

Entry of the virus into the cell proceeds via the loss of VP2. Low pH triggers a major conformational change in VP5 which enables the penetration of the membrane via the extended helical structure. The slight distortion of the VP7 lattice into which VP5 fits weakens the attachment of VP5 and may facilitate ejection of the replication competent core particle into the cell to initiate production of RNA and progeny virus.

## Abbreviations

BTV: bluetongue virus
dsRNA: double-stranded RNA
OPPF: Oxford Protein Production Facility
r.m.s.d.: root mean square deviation
SHP: Structure Homology Program
